# Cerebellar radiological abnormalities in children with neurofibromatosis type 1: part 2 - a neuroimaging natural history study with clinical correlations

**DOI:** 10.1186/s40673-018-0092-z

**Published:** 2018-10-30

**Authors:** Michael S. Salman, Shakhawat Hossain, Samantha Gorun, Lina Alqublan, Martin Bunge, Katya Rozovsky

**Affiliations:** 1 Section of Pediatric Neurology, Winnipeg Children’s Hospital and Department of Pediatrics and Child Health, Max Rady College of Medicine, Rady Faculty of Health Sciences, University of Manitoba, AE 308, 820 Sherbrook Street, Winnipeg, MB R3A 1R9 Canada; 2 Department of Mathematics and Statistics, University of Winnipeg, Winnipeg, MB Canada; 3 Department of Radiology, Max Rady College of Medicine, Rady Faculty of Health Sciences, University of Manitoba, Winnipeg, MB Canada; 4 Present Address: Department of Radiology, King Fahad Armed Forces Hospital, Jeddah, Western region Saudi Arabia; 5 Section of Pediatric Radiology, Department of Radiology, Max Rady College of Medicine, Rady Faculty of Health Sciences, University of Manitoba, Winnipeg, MB Canada

**Keywords:** Cerebellum, Neurofibromatosis type 1, Focal abnormal signal intensity, Natural history, Pediatrics

## Abstract

**Background:**

Focal abnormal signal intensities (FASI) on brain MRI occur commonly in patients with neurofibromatosis type 1 (NF1). The natural history of cerebellar FASI and their correlation with clinical features have not been studied comprehensively. Our aims are to describe the natural history of cerebellar FASI on repeat MRI scans and correlate the findings with the clinical features in children with NF1 and cerebellar FASI.

**Method:**

A retrospective review of 226 brain MRI scans and hospital charts was performed in 50 patients with cerebellar FASI, who were diagnosed with NF1 during their childhood between 1999 and 2008.

**Results:**

Mean age at the end of the study period was 16.1 years. There were 27 males. Mean duration of clinical follow up was 10.1 years. Mean duration between the first and the last MRI was 6.6 years (*n* = 36, SD: 2.8 years). FASI were rarely confined to the cerebellum. The number of FASI was highest in early childhood and decreased significantly on subsequent MRI scans in most brain regions with the exception of the cerebrum, where a fewer number of patients with a smaller number of FASI were seen. Four patterns of change in FASI size over time were determined, none correlated with the clinical features.

**Conclusions:**

In patients with NF1, the natural history of FASI including their number, age at onset, rate of size changes, and resolution if any, varies by brain region. FASI patterns of change over time showed no clinical correlate.

## Background

Neurofibromatosis type 1 (NF1) is a common neurocutaneous disorder. Its estimated incidence is 1/3500 live births [1]. At least two of seven major criteria are required for diagnosis including: Two neurofibromas or one plexiform neurofibroma, an optic glioma, two Lisch nodules, a first-degree relative with NF1, six café-au-lait spots, axillary or inguinal freckling, or a distinctive osseous lesion [1]. Patients with NF1 are at increased risk of developing optic pathways gliomas and other tumors [2–5].

NF1 may be associated with cognitive dysfunction. Visuospatial and fine motor deficits occur commonly [6, 7]. Learning disability has been reported between 35 and 65% on children with NF1 [6]. Attention-deficit hyperactivity disorder is also reported commonly [6].

The most common abnormalities on brain MRI are regions of increased signal visible on T2-weighted images i.e., focal abnormal signal intensities (FASI), previously labelled by some investigators as unidentified bright objects (UBO). FASI is present in up to 93% pediatric patients with NF1 [3, 8, 9]. Typically, they increase in number and size in early childhood and then disappear in general during adolescence [10]. They are very uncommon in patients older than twenty years [9, 10]. FASI are most common in the deep gray matter, brainstem, cerebellum, and at times cerebral hemispheres [9–11]. Most studies investigating the clinical significance of FASI agree that there is no correlation between neurological deficits and the presence of FASI or their total number [12]. FASI located in the thalamus was found to be associated with cognitive impairment by some investigators [8, 13].

The cerebellum is important for non-motor, cognitive, and motor tasks [14]. Cognitive and neuropsychiatric disorders including developmental delay, learning disabilities, and behavioral difficulties have been reported in children with cerebellar diseases [14]. We hypothesized that children with NF1 and cerebellar FASI, and especially in those showing a pattern of enlarging FASI size or the new appearance FASI over time, were more likely to have cerebellar motor signs and developmental delay and/ or learning disabilities.

The aims of this project are to: Describe the neuroradiological features in children with NF1 and cerebellar FASI on MRI, determine the natural history of FASI on repeat MRI, and correlate them with the clinical features of the patients, especially developmental delay, learning disabilities, and examination findings.

## Methods

We selected pediatric patients less than 17 years old with NF1 if they had cerebellar abnormalities mentioned on their neuroimaging reports during the years 1999–2008. Clinical information available until 2013 was extracted from their hospital chart at Winnipeg Children’s Hospital. Ethical approval for the study was given by the Research Ethics Board of the University of Manitoba.

Inclusion criteria for the study were: [1] Patients had NF1 and were less than 17 years old during 1999 and 2008, and [2] cerebellar abnormalities were reported on their MRI. Exclusion criteria were: [1] Patients with solid central nervous system tumors unrelated to NF1 or if they had other unrelated brain disease e.g. demyelinating diseases, [2] trauma or brain malformations unrelated to NF1, [3] patients who had brain radiotherapy or chemotherapy before their first brain MRI.

Data extracted from the hospital chart consisted of demographic information, birth and family history, symptoms, developmental milestones, learning disabilities, and physical exam findings especially eye and central nervous system examination abnormalities.

Brain MRI was acquired on 1.5 or 3 Tesla MRI scanner (GE) using standardized protocol with sagittal T1-weighted, axial and coronal T2-weighted, and axial and coronal fluid-attenuated inversion recovery (FLAIR) images. Other imaging sequences were added as needed including T2*, DWI, ADC (apparent diffusion coefficients) maps, fast spoiled gradient echo (FSPGR) images, and MRA. Contrast with Gadolinium was given at the discretion of the radiologist. All brain MRI images available that were completed by June 2014 were reviewed independently by two pediatric radiologists with expertise in neuroimaging. Disagreements were resolved by consensus. Further details on this cohort is available in another study (see part one of this investigation).

Age at the time of each MRI scan, structural and signal abnormalities in the cerebellum, brainstem, and supratentorial structures (i.e., cortex and white matter, basal ganglia, thalami, hypothalamus, and optic nerves/ chiasm) were recorded, as well as the presence of cerebellar hypoplasia or atrophy. Optic pathways gliomas were diagnosed when these structures were enlarged on MRI. Detailed information was collected on FASI on FLAIR images including their total number, locations, diameter (defined as a straight line passing through their center and thus representing their maximum length), uptake of contrast on T1-weighted images, change in size or signal over time (if any), malignant transformation (if any), and mass effect.

The natural history of FASI on repeat MRI scans, i.e. the change in the lesions’ size over time was determined individually in each of the following five locations: cerebellum, brainstem (midbrain, pons, medulla, cerebellar and cerebral peduncles), thalamus/ hypothalamus, basal ganglia/ internal capsule (these structures were combined since lesions in the internal capsules tended to involve the basal ganglia and it was difficult to separate the two, as was also reported previously) [15], and cerebrum (cortex, subcortical and periventricular white matter, hippocampus, corpus callosum, and fornix).

The change in FASI size was allocated to one of the following numerical categories: 1) if full resolution occurred, 2) if FASI became smaller or if FASI remained the same size but another FASI was now smaller/ resolved within the same location, 3) if FASI was unchanged in size, 4) if variable changes were seen in FASI within the same location, i.e. FASI became smaller, unchanged, or resolved but another FASI in the same location has increased in size, 5) if FASI increased in size, or if FASI remained the same size but another FASI has increased in size within the same location, 6) if a new FASI was seen irrespective of changes seen in other FASI in the same location, and 7) if FASI was surgically removed. Different patterns of changes in the size of FASI over time in each of the five brain locations were described. These patterns of changes over time were compared across the five brain locations to investigate if the patterns of changes were concordant (i.e., congruent) or vary across different brain regions.

Mean and median were used to describe normally distributed and skewed data, respectively. Paired t-test was performed to investigate if the difference between the number of FASI at baseline scan and the number of FASI at last scan in each brain region was significant.

For each patient, the longest diameter of FASI was selected irrespective of its location. The change in FASI’s longest diameter between the first and last MRI scan was investigated similarly using paired t-test to ascertain if there was a tendency for FASI's longest diameter to decrease with age in individual patients. Pearson’s chi-squared test (or Fisher’s exact test if necessary) was performed to investigate the association of a single location, combination of locations, and patterns (change with time) of FASI with the clinical features (developmental delay, learning disabilities, headache, ADHD, seizures, family history of NF1) and other categorical variables i.e., clinical signs (visual acuity, visual fields, funduscopy, strabismus, nystagmus, tone, strength, reflexes, gait, and wheelchair use). Binary logistic and Poisson regression methods were used to model binary (e.g., various brain locations) and count (e.g., number of FASI at first and last scan) responses, respectively with age, symptoms, and signs. To account for correlations within the patients’ responses, generalized linear mixed model was conducted to investigate the relationship between age and FASI change in pattern on repeat MRI in the five brain locations. To facilitate this analysis, changes in FASI with time were grouped into three categories (improved [i.e., resolution or smaller], remained the same, or worsened [i.e., larger or new FASI developed]) and age into four categories (1–4.9, 5–9.9, 10–13.9, and 14 years and older). All analyses were performed in Statistical Analysis Systems (SAS) 9.2 (SAS Institute, Cary NC). Significance was defined when *p*-value (p) was less than 0.05.

## Results

Fifty patients fulfilling the inclusion criteria were identified. Their mean age was 16.1 years (SD: 5.5 years) at the end of the study period. There were 27 males and 23 females. Mean duration of follow up was 10.1 years (SD: 5.9 years). Four died from malignant tumours. Neurofibromas occurred in 27 patients, optic pathways gliomas in 21, learning disability in 25, and developmental delay in 20 patients. Positive family history of NF1 was present in 24 patients. Decreased visual acuity was reported in 9, optic disc pallor in 18, and decreased tone in 9 patients. Other clinical features on the cohort are available in part 1 of this investigation.

### Neuroimaging

Some brain MRI scans during the early study period were not available for review. Therefore in some patients, the first MRI scan reviewed and discussed in this paper may not have been their first MRI scan. Two hundred and twenty six brain MRI scans were reviewed. Fourteen patients had a single brain MRI scan, the remainder (*N* = 36) had at least one repeat MRI scan. The median number of scans reviewed per patient was 3 (minimum: 1, maximum: 16, interquartile range: 5). Mean age of the patients on the first MRI scan reviewed was 7.8 years (*N* = 50, SD: 4.5 years, median: 7.3 years, minimum: 1.2, maximum: 18.2 years), where SD is the standard deviation. Mean age of the patients on the last MRI was 13.9 years (*N* = 36, SD: 5.7 years, median: 13.6 years, minimum: 3.2, maximum: 28.8 years). There was a significant difference between the ages at the first and last MRI scans (*p* <  0.0001). Mean duration between the first and last MRI scans was 6.6 years (*N* = 36, SD: 2.8 years, median: 6.6 years, minimum: 0.4, maximum: 12.9 years).

### The natural history of the number of FASI in different brain regions

Table 1 shows the number of patients with FASI in different brain locations on the first and subsequent MRI scans reviewed. Total FASI count is also displayed. FASI was most commonly seen in the brainstem and basal ganglia in addition to the cerebellum. One patient had two basal ganglia FASI on the first scan but developed FASI in the middle cerebellar peduncle on the second MRI. The rest of the patients had cerebellar FASI on the first MRI scan reviewed. In many patients, FASI was present concurrently in multiple brain locations.

**Table 1:**
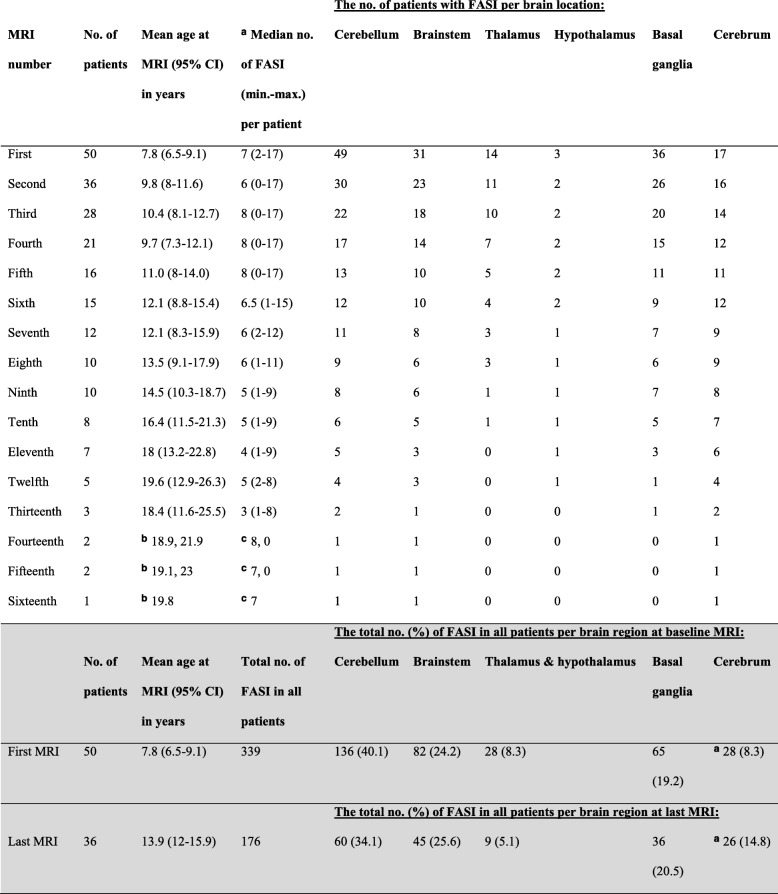
The number (no.) of patients with focal abnormal signal intensities (FASI) in different brain locations on the first and subsequent MRI scans is shown. The total no. of FASI on the first and last MRI is also shown for comparison (shaded area)

^a^The patient with the numerous cerebral lesions is excluded

^b^actual ages

^c^actual numbers since there were only 1 or 2 patients, min.: minimum, max.: maximum

**Fig. 1:**
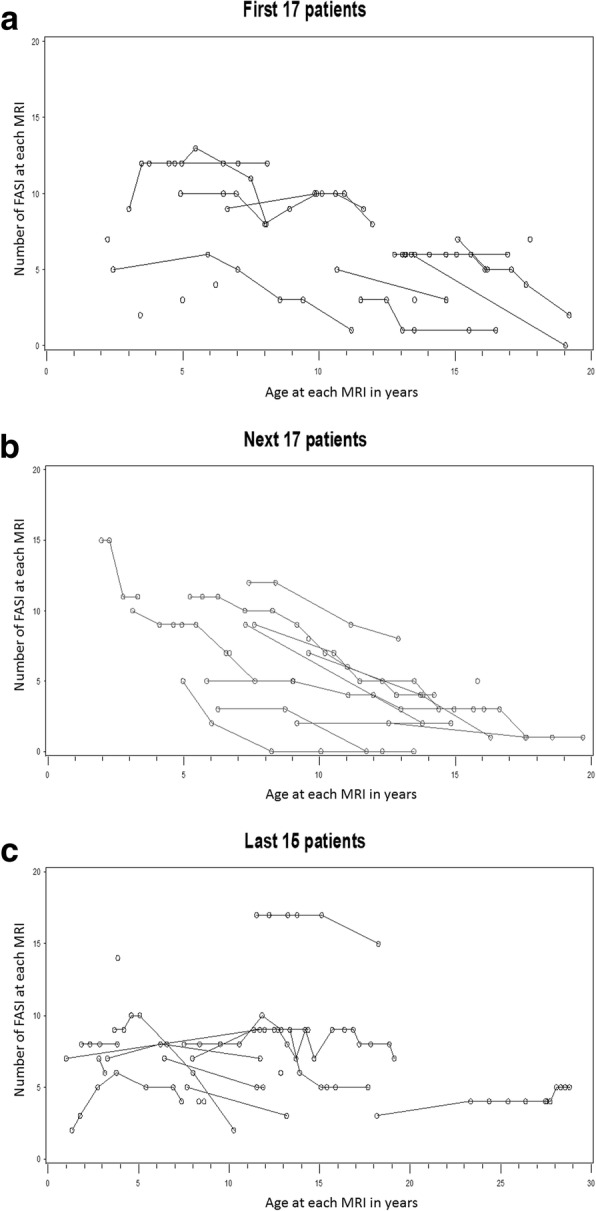
The graphs show the number of focal abnormal signal intesities (FASI) at baseline MRI and on repeat MRI scans in patients with NF1 plotted against the age at which each MRI scan was done. Each line connects individual patients. The 49 patients are plotted sequentially with the first 17 patients displayed in (**a**), the next 17 patients in (**b**), and the last 15 patients in (**c**). Indiviual patients, who did not have repeat MRI are shown as lone circles. Patient #54, who had numerous cerebral FASI is excluded. The total number of FASI decreased significantly with increasing age

**Fig. 2:**
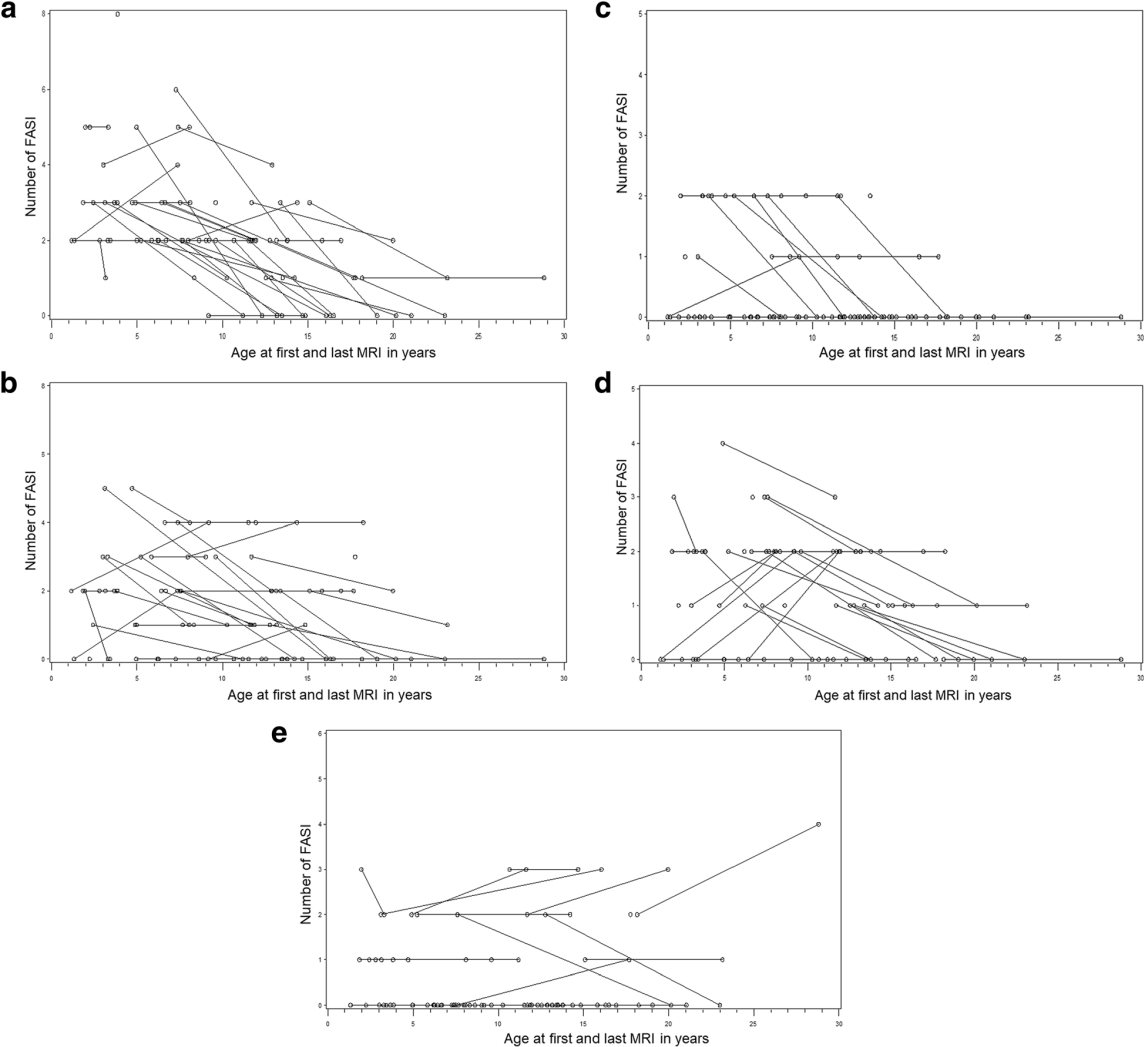
The graphs show the number of focal abnormal signal intesities (FASI) at baseline and last MRI scans in patients with NF1 plotted against the age at which each MRI scan was done. Five brain locations are shown - (**a**) cerebellum, (**b**) brainstem, (**c**) thalamus and hypothalamus, (**d**) basal ganglia and internal capsule, and E: cerebrum. Each line connects individual patients. Patients who only had one MRI are shown as lone circles. Patient #54, who had numerous cerebral lesions is excluded in (**e**). The number of FASI decreased significantly as age increased in all brain regions with the exception of the cerebral region

Tables 2 and 3 shows the mean and median number of FASI in different brain locations on the first and last MRI scans. A single patient (#54) had widespread numerous FASI in the cortex and subcortical white matter of both cerebral hemispheres. To minimize extreme skewness of the data, we excluded this patient from the data analysis involving cerebral FASI counts (Tables 1, 2 and 3). The number of FASI decreased significantly in general and in several brain locations except the basal ganglia/internal capsule and cerebral hemispheres (Table 2). To account for correlations in the number of FASI on repeat MRI scans, generalized linear mixed model with Poisson link was conducted to investigate the effect of age on the number of FASI in several brain locations. The number of FASI decreased significantly as age increased in all brain regions with the exception of the cerebral region (Table 3, Figs. 1 and 2). The estimated rate of decrease in the number of FASI was larger for the thalamus and cerebellum in comparison to the basal ganglia/ internal capsule.

**Table 2:** The mean and median number (No.) of focal abnormal signal intensities (FASI) in different brain locations on the first and last MRI in patients with repeat MRI

Brain location	No. of patients at baseline	No. of FASI on first MRI		No. of patients at last MRI	No. of FASI on last MRI		*p-value
		Mean (SD)	Median (min.-max.)		Mean (SD)	Median (min.-max.)	
Overall	35	7.3 (3.5)	7 (2–17)	35	4.8 (3.8)	5 (0–15)	< 0.0001
Infratentorial	36	3.5 (2)	3 (0–10)	36	2.1 (2.3)	1.5 (0–10)	0.0002
Supratentorial	35	3.8 (2.2)	4 (0–8)	35	2.7 (2)	3 (0–7)	0.001
Cerebellum	36	2.8 (1.6)	2.5 (0–9)	36	1.7 (1.9)	1 (0–9)	< 0.0001
Brainstem	36	1.9 (1.5)	2 (0–5)	36	1.3 (1.4)	1 (0–4)	0.007
Thalamus/hypothalamus	36	0.5 (0.8)	0 (0–2)	36	0.3 (0.6)	0 (0–2)	0.031
Basal ganglia/internal capsule	36	1.3 (1.1)	1 (0–4)	36	1 (1)	1 (0–3)	0.106
Cerebrum	35	0.7 (1)	0 (0–3)	35	0.7 (1.2)	0 (0–4)	0.812

*Significance level on paired t-test is the difference in the mean number of FASI between the first and last MRI in patients who had a repeat MRI, min.: minimum, max.: maximum

**Table 3:** The effect of increasing age on the number of focal abnormal signal intensities (FASI) in different brain locations in patients with NF1

Brain location	Estimate of age effect of FASI number	^a^DF (numerator, denominator)	F-value	*P*-value
^b^Overall	−0.05503	1166	44.99	< 0.0001
Infratentorial	− 0.06517	1,35	18.78	0.0001
Supratentorial	−0.03646	1,34	7.27	0.011
Cerebellum	−0.06929	1,35	18.38	0.0001
Brainstem	−0.05143	1,35	6.49	0.015
Thalamus/ hypothalamus	−0.1023	1,35	6.51	0.015
Basal ganglia/internal capsule	−0.04034	1,35	4.23	0.047
Cerebrum	0.02318	1,34	0.67	0.418

^a^DF: degrees of freedom

^b^Data from all MRI repeats in each patient were used for this analysis. For more specific brain locations (below), data from the first and last MRI were used

Univariate regression analysis revealed that there were significant associations between the age of the patients at the first MRI and the number of FASI in the infratentorial region and cerebellum (*N* = 50, *p* = 0.048 and *N* = 50, *p* = 0.048, respectively). As age on the first scan increased, the number of FASI decreased in the infratentorial and cerebellar regions. In addition, there were significant associations between the age of the patients at the last MRI and each of the following: 1) total number of FASI, 2) number of FASI in the supratentorial region, and 3) number of FASI in the basal ganglia/ internal capsule (*N* = 35, *p* = 0.033; *N* = 35, *p* = 0.041; and *N* = 36, *p* = 0.009, respectively). As age on the last scan increased, the total number of FASI decreased in general. The number of FASI also decreased significantly in the supratentorial region and more specifically in the basal ganglia/ internal capsule region. There was a trend for the number of FASI in the cerebellum on the last MRI to also decrease with age at last MRI scan (N = 36, *p* = 0.054). There were no significant associations between the total or regional number of FASI on the first MRI and age of the patients at: symptom onset, first clinic visit, or the end of the study.

### The association between maternal age at conception and the number of FASI

A significant relationship was found between maternal age at conception and the total number of FASI at last MRI (*p* = 0.044). With every one-year increase in maternal age at conception, the expected total number of FASI at last MRI was 3.01% lower. Associations between the total number of FASI at baseline MRI and clinical features can be found in part 1 of this investigation.

### The longest diameter of FASI

The longest diameter of FASI among our patients in the first scan had a median length of 1.6 cm (*N* = 49, minimum: 0.8, maximum: 5.6 cm). The image was degraded by motion artifact in one patient, therefore measurement was not possible. FASI with the longest diameter were mostly located in the cerebellum on the first MRI (in 22 of 49 patients). The longest diameter of FASI among our patients in the last scan had a median length of also 1.6 cm (*N* = 31, minimum: 0.7, maximum: 5.0 cm). FASI with the longest diameter were mostly also located in the cerebellum (in 11 of 31 patients who had repeat MRI and persisting FASI on their last MRI). FASI with diameters longer than 2.5 cm were mostly caused by several confluent FASI, which could not be measured individually. Paired t-test showed that there was no significant change in FASI’s longest diameter between the first and last MRI (N = 31, *p* = 0.665).

### The natural history of FASI’s size

**Fig. 3:**
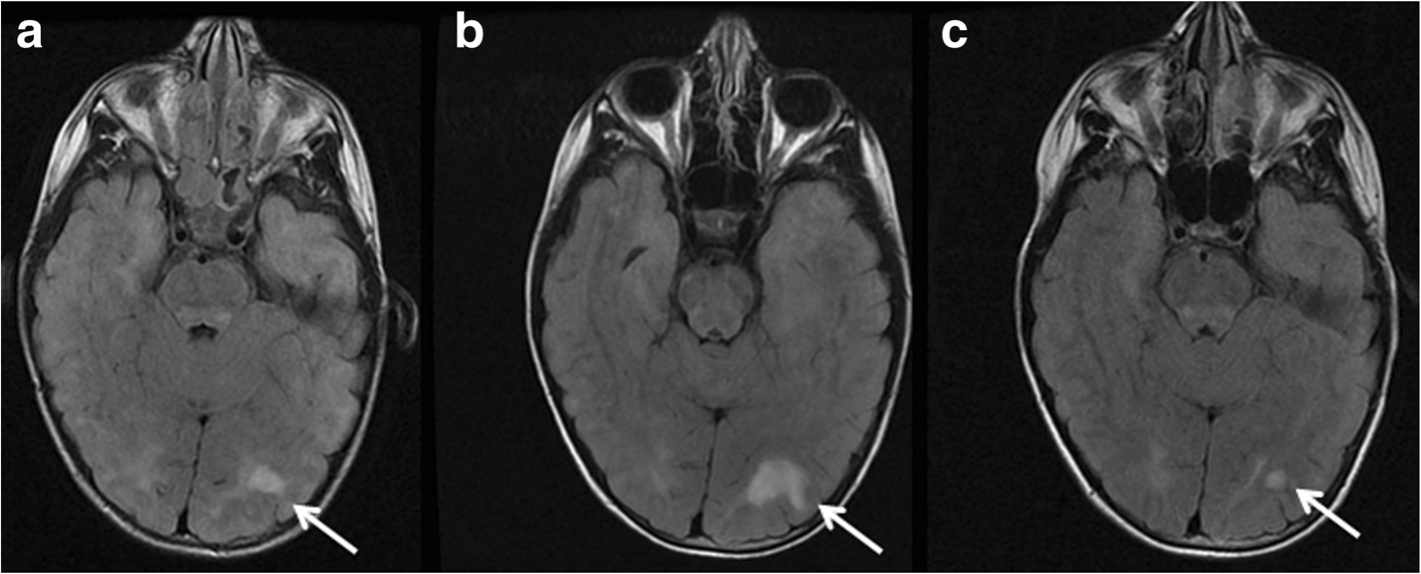
The figure shows pattern B (see text) on axial brain MRI in a patient with NF1. Bilateral occipital abnormal signal intensities are evident. The left occipital abnormal signal intensity (arrow) increased in size from age 4 years (**a**) to age 7 years (**b**). It then decreased in size and stabilized at age 9 years (**c**)

**Fig. 4:**
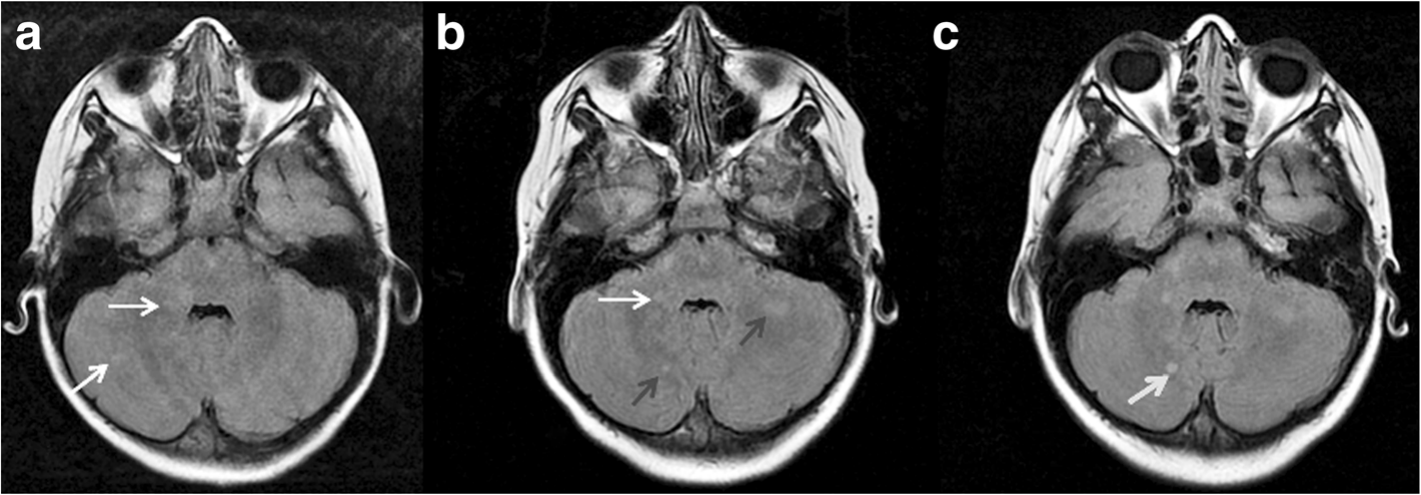
The figure shows pattern D (see text) on axial MRI in a patient with NF1. Two small right cerebellar abnormal signal intensities (arrows) are seen at 8 years (**a**). New right and left cerebellar abnormal signal intensities (dark oblique arrows) developed at age 11 years. One of the older abnormal signal intensities is also shown (horizontal arrow) (**b**). The right cerebellar abnormal signal intensity increased in size (arrow) at age 13 years (**c**)

Changes in the size of FASI over time among patients who had repeat MRI scans, fell into four distinct patterns irrespective of their brain location. These patterns were grouped as follows: In patients showing pattern A, FASI either remained unchanged or decreased in size then remained unchanged on repeat MRI. In patients with pattern B, FASI were either stable or decreasing in size but then enlarged in size or a new lesion(s) developed. Subsequently, FASI either disappeared, or decreased in size, or remained the same size (Fig. 3). In patients with pattern C, FASI either decreased in size or disappeared. Finally, in patients with pattern D, FASI became larger in size following a period when their size remained stable or was decreasing (Fig. 4). Table 4 shows the number of patients with each of these patterns and for each of the five brain locations involved. Many patients with FASI in the cerebellar, brainstem, thalamus/ hypothalamus regions followed patterns A or C, i.e. the relatively non-aggressive patterns, followed closely by pattern B. A small minority of patients followed pattern D (though none in the thalamus/ hypothalamus group), which is a more aggressive pattern.

**Table 4:** Patterns of changes in size in focal abnormal signal intensities (FASI) on brain MRI over time in patients with NF1 are displayed. The number (%) of patients for each pattern and brain location is shown

^a^Pattern type	Cerebellum (*n* = 35)	Brainstem (*n* = 28)	Thalamus/ Hypothalamus (*n* = 14)	Basal ganglia/ Internal capsule (*n* = 28)	Cerebral (*n* = 17)
A	6 (17.1)	10 (35.7)	6 (42.9)	10 (35.7)	4 (23.5)
B	12 (34.3)	9 (32.1)	3 (21.4)	12 (42.9)	7 (41.2)
C	14 (40)	7 (25)	5 (35.7)	4 (14.3)	1 (5.9)
D	3 (8.6)	2 (7.1)	0	2 (7.1)	5 (29.4)

^a^Pattern types:

A: FASI has remained stable in size or has decreased in size before stabilizing again

B: FASI has been increasing in size or a new FASI has developed, which eventually either got smaller, stabilized or disappeared. FASI may have been preceded by a period of stability in size or a decrease in size before the enlargement occurred

C: FASI has been getting smaller in size or has disappeared

D: FASI has been increasing in size or a new FASI has developed. These changes may have been preceded by a period of stability in size or a decrease in size before the enlargement occurred

On the other hand, many patients with FASI in the basal ganglia/ internal capsule, and cerebral regions followed pattern B with a significant proportion of patients with FASI in the cerebral region following pattern D. A few patients with FASI in the basal ganglia/ internal capsule regions followed pattern D, with many following pattern A. There was a trend for the basal ganglia (*p* = 0.057) and cerebral patterns (*p* = 0.053) to be related to the age of the patients at first clinic visit, whereby patterns B and D tended to be seen in the patients that were in the youngest age group on their first clinic visit (0–0.9 year versus 1–1.9, 2–4.9, and 5 years or older).

Patients were split into groups based on their age because the ages at baseline and repeat MRI were heterogeneous among patients. Results from the generalized linear mixed model showed that changes in FASI’s pattern occurring in the cerebellum and basal ganglia/ internal capsule regions only, were significantly related to age (*p* = 0.029 and *p* = 0.027, respectively). Specifically, the odds of FASI pattern changing from *resolution/getting smaller to larger/new FASI in the cerebellum* on repeat MRI in children in the 1–5 years age group was 3% (95% CI: 0.3–32.6) lower than the odds for children ≥14 years old. Similarly, the odds of FASI pattern changing from *stable in size to larger/new FASI in the cerebellum* on repeat MRI in children in the 1–5 years age group was 19.3% (95% CI: 5.0–74.0) lower than the odds for children ≥14 years old.

As for the basal ganglia/ internal capsule region, the odds of FASI pattern changing from *resolution/getting smaller to larger/new FASI* on repeat MRI in children in the 1–5 years age group was 6.1% (95% CI: 0.7–55.9) lower than the odds for children ≥14 years old. Similarly, the odds of FASI pattern changing from *stable in size to larger/new FASI* on repeat MRI in children in the 1–5 years age group was 12.3% (95% CI: 2.0–77.3) lower than the odds for children ≥14 years old. In addition, the odds of FASI pattern changing from *resolution/getting smaller to larger/new FASI* on repeat MRI in children in the 5–10 years age group was 11.2% (95% CI: 1.9–65.3) lower than the odds for children ≥14 years old. Similarly, the odds of FASI pattern changing from *stable in size to larger/new FASI* on repeat MRI in children in the 5–10 years age group was 10.5% (95% CI: 2.0–55.7) lower than the odds for children ≥14 years old.

To further investigate the effect of age on FASI patterns in different brain locations, we created two pattern groups and three or four age groups to facilitate statistical analysis. The first pattern group was a non-aggressive FASI group involving patients with patterns A and C. The second group had a more aggressive pattern of FASI involving patients with patterns B and D. There was a significant relationship only between cerebellar FASI pattern and the age of patients at the end of the study. Patterns B and D were significantly more common in the youngest age group (6–11 years at the end of the study) in comparison with the older age groups (12–17 years and 18 years or older, *p* = 0.012).

There was no significant relationship between the sex of the patients and FASI patterns or their locations.

### Congruity of FASI pattern (size changes with time) across different brain regions

FASI patterns were fully congruent i.e., the same patterns occurred in all brain regions involved, in 8 patients (23.53%). There were another 8 patients (23.53%) whose FASI patterns were congruent in at least 3 brain regions. The number of patients with at least 3 different patterns in their affected brain regions was 8 (23.53%). Table 5 shows the different patterns across the five brain regions in each of the 34 patients who had a repeat MRI and had FASI in at least 2 different brain locations.

**Table 5:**
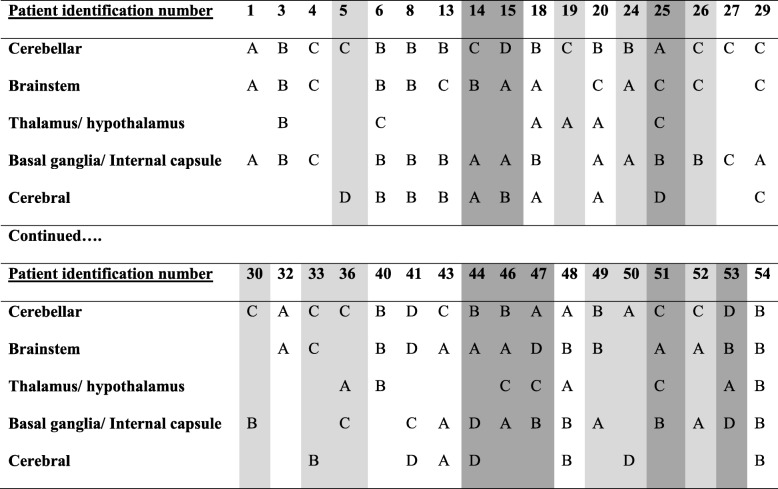
^a^ Patterns of changes in size in focal abnormal signal intensities (FASI) on brain MRI over time in patients with NF1 across different brain regions are displayed in each patient. Patients with more (light gray) and most (dark gray) incongruent patterns across different brain locations are highlighted

^a^Pattern types:

A: FASI has remained stable in size or has decreased in size before stabilizing again

B: FASI has been increasing in size or a new FASI has developed, which eventually either got smaller, stabilized or disappeared. FASI may have been preceded by a period of stability in size or a decrease in size before the enlargement occurred

C: FASI has been getting smaller in size or has disappeared

D: FASI has been increasing in size or a new FASI has developed. These changes may have been preceded by a period of stability in size or a decrease in size before the enlargement occurred

### The relationship between the clinical features and FASI patterns (size changes with time)

Univariate analysis and multiple logistic regression analysis showed that there is no significant relationship between FASI patterns (size change with time) and symptoms or signs. For this part of the analysis, patients with patterns A and C were combined to form one group, while patients with patterns B and D formed the other group to facilitate valid statistical analysis.

### Mass effect and contrast enhancement of FASI on repeat MRI

Table 6 shows the number of patients with FASI that displayed contrast enhancement and mass effect on repeat MRI. Details on these patients and further discussion on these atypical FASI features can be found in part 1 of this investigation.

**Table 6:** The number (No.) of patients with a) optic pathways gliomas, b) mass effect from focal abnormal signal intensities (FASI), and c) contrast enhancement of FASI on their first and subsequent MRI scans is shown

MRI number	No. of patients	Median age at MRI (min.-max.) in years	^a^No. of patients with optic pathways gliomas	The no. of patients with FASI displaying:	
				^a^Mass effect	^b^Contrast enhancement
First	50	7.3 (1.2–18.2)	18	3	5
Second	36	9.4 (1.8–23.4)	17	2	3
Third	28	11 (2.8–24.4)	16	2	4
Fourth	21	10.1 (3.3–25.4)	16	2	4
Fifth	16	10.6 (4.4–26.4)	11	1	2
Sixth	15	11.2 (5.4–27.5)	11	0	4
Seventh	12	10.2 (5.8–27.6)	10	0	3
Eighth	10	12.5 (7.4–27.7)	9	0	4
Ninth	10	13.7 (8.1–28.1)	9	0	4
Tenth	8	15.9 (9.2–28.3)	7	0	4
Eleventh	7	16.9 (13–28.6)	6	0	3
Twelfth	5	17.7 (15–28.8)	4	1	3
Thirteenth	3	17.8 (16.1–21.4)	2	0	2
Fourteenth	2	20.4 (18.9–21.9)	1	0	1
Fifteenth	2	21.1 (19.1–23)	1	0	1
Sixteenth	1	19.9	1	0	1

^a^In one patient the information could not be obtained since relevant images were degraded by motion artifacts

^b^In 6 patients, no contrast was given in one of their scans (further details and discussion on these patients can be found in part 1 of this investigation), min.: minimum, max.: maximum

### Optic pathways gliomas and their outcome on repeat MRI

Table 6 shows the number of patients with optic nerve(s) and/ or chiasm gliomas on the first and subsequent MRI scans. Of the 50 patients, 21 (42%) had optic pathways gliomas at any scan. Of those 21 patients, there was resolution of these gliomas in 2, persistence in 17, while the outcome was unknown in 2 since they did not have a repeat scan. Correlation with clinical features can be found in part 1 of this investigation.

## Discussion

FASI is highly prevalent in patients with NF1 [8, 9, 16], and up to 84% of these patients have infratentorial FASI [9, 10, 17–19]. The nature of FASI remains unknown but increased fluid within the myelin associated with hyperplastic or dysplastic glial proliferation has been suggested [20–22]. Our investigation focuses on the natural history of FASI and their clinical correlate in NF1 patients who had cerebellar FASI during their childhood. Since the cerebellum participates in a wide range of motor and non-motor functions [14], we selected patients with cerebellar FASI to investigate whether FASI presence and their size change with age is associated with any clinical features or consequences.

The presence, change in size, or persistence of cerebellar FASI was not associated with cerebellar motor signs. Prior studies have reported the absence of cerebellar signs in patients with NF1 irrespective of the presence of FASI [8, 13, 16, 23, 24]. Therefore, if a patient with NF1 develops ataxia, then this should alert the clinician to the presence of a cerebellar, brainstem or spinal cord tumor, or a lesion compressing the spinal cord [1, 4].

The number of FASI is highest in early childhood and decreases with age [10, 11, 18, 25, 26], as we also found. Their number decreased significantly on repeat MRI in all brain regions with the exception of the basal ganglia and cerebral hemispheres. When age was taken into account, the number of FASI in the basal ganglia also showed a decrease with age.

The number of patients with cerebral FASI and the number of FASI in the cerebral hemispheres were not particularly high at any age in our cohort but they seemed to persist. This is in agreement with findings from a prior study of 19 patients with cerebral hemispheric FASI who were followed up for a shorter period of 1–2 years [10], and another study from the same group of investigators, who confirmed the findings on long term follow up. They reported that FASI in the brainstem, thalamus, and basal ganglia tended to resolve while those in the cerebral hemispheres and deep white matter were mostly stable. The mean age of their 18 NF1 patients was 29.4 (SD: 2.3) years [25]. Indeed, when increasing age in individual patients was factored in our analysis; the number of FASI in the cerebral hemispheres only, was not apparently related to age. The reason is unknown. Hormonal and genetic influences are suspected or they may have different pathological basis [10, 26].

In contrast with our findings, a longitudinal study of 12 children with NF1 showed that FASI increased during adolescence in all brain regions in some patients, except for the cerebellar hemispheres where it decreased [15]. Their patients were selected after the exclusion of optic gliomas, seizures, and uncorrectable visual impairment, which may explain the difference in their findings. A study with more patients focusing on cerebral FASI over a longer time period of observation into adulthood is warranted to investigate cerebral FASI further.

The rate at which the number of FASI decreased with age varied, with the thalami and cerebellum showing the fastest decline and the basal ganglia/ internal capsule showing the slowest decline. When a cross sectional investigation of the age of the patients at the first and last MRI scans (as opposed to the longitudinal change in the number of FASI in each individual patient with age as described above) were each correlated with the number of FASI in our cohort, similar results were obtained at specific brain locations. The number of FASI in the cerebellum decreased as the age of different patients on the first MRI increased while the number of FASI in the basal ganglia/ internal capsule decreased as the age of various patients increased on the last MRI scans; suggesting that FASI rate of decline with time is age-dependent and location-specific i.e., younger age for the cerebellum and older age for the basal ganglia/ internal capsule.

Overall, FASI with the longest diameter tended to occur most commonly in the cerebellum on the first and last MRI scans. This finding may have arisen because of selection bias since patients with NF1 and cerebellar FASI were specifically selected for our study. Alternatively, we speculate that the anatomy and structure of the cerebellum in NF1 may allow a larger size of FASI to develop or perhaps cerebellar FASI may have a different pathology.

Although the number of FASI decreases with age and in most brain regions in general; individually their size can decrease, increase, or remain stable on repeat MRI. When a single FASI with the longest diameter was selected from the first and last MRI scans irrespective of its brain location in each patient, the median length of this measurement was the same between the two MRI scans in our cohort. This suggests that in patients with persisting FASI, the maximum potential diameter that FASI can attain anywhere in the brain, is age-independent. In one study, the maximum diameter of FASI was 2.5 cm [18]. In some instances in our study, it was not possible to measure the maximum diameter of individual FASI, which accounted for lengths > 2.5 cm since a few FASI coalesced. Other investigators also found that determining the outer boundary of some FASI can be difficult since some are not a discrete lesion [10].

FASI followed four patterns of size changes on repeat MRI scans. FASI in the cerebellar, brainstem, thalamus/ hypothalamus regions tended to follow relatively non-aggressive patterns. On the other hand, many patients with FASI in the basal ganglia/ internal capsule, and cerebral regions tended to show more aggressive patterns, since many showed an increase in lesion size or the appearance of new lesions, especially in the cerebrum as others have also reported [10, 15, 26]. Although congruity in these FASI patterns across various brain regions was noted in several instances, this was not uniformly seen. Therefore, FASI pattern of size change can evolve differently across the various brain regions with time.

Age influenced the changes seen in FASI size on repeat MRI scans. This was only significant for the cerebellar and basal ganglia/ internal capsule regions, whereby the odds of developing an aggressive pattern (patterns B and D i.e., enlargement in an existing FASI or the development of a new FASI), was smaller in early childhood in comparison to patients 14 years or older. More specifically, a more aggressive pattern of FASI (pattern B and D) developing in the cerebellum seems to occur at the 6–11 years age group in comparison with older children. The reason is unknown. However, this pattern of FASI changes was different for the basal ganglia and cerebral regions, since a trend was found for a more aggressive pattern to subsequently develop in patients who present in their first year of life to clinic for an assessment regarding NF1. Perhaps these patients have a different phenotype of NF1 since they present to medical attention very early in life. Further studies are needed to replicate these findings. However, FASI patterns i.e. changes in their size over time was not associated with any of the clinical symptoms and signs, consistent with the apparently benign nature of FASI.

In summary, it appears that in patients with NF1, various brain regions are affected differently by FASI since the age at which they develop, where they develop, the longest diameter they attain, their number and its rate of change with age, their natural history i.e., the size they follow over time, and their resolution (or persistence) varies considerably by brain region. Genetics, epigenetics, structural, and possibly hormonal factors are suspected.

Limitations of our study include incomplete ascertainment of patients, variable follow up durations, missing information, variable number of MRI scans repeats, variable ages at which MRI were repeated, non-uniform MRI magnetic strength and protocols used, inaccurate diameter measurement when FASI were ill-defined, and lack of comprehensive cognitive assessment. In addition, the patterns of FASI described are dependent on the age of the patients at the time of the MRI scan, number of MRI scans they had, and the time intervals between the MRI scans.

## Conclusions

Patients with NF1 and cerebellar FASI do not have cerebellar motor symptoms or signs despite the changes in FASI sizes with age. The number of FASI was highest in early childhood and their number tended to decrease everywhere with age with the exception of the cerebrum, where a fewer number of patients with a smaller number of persisting FASI were seen. The natural history of FASI besides their number and including the age of their onset, size change, and resolution if any, varies by brain region in patients with NF1 and cerebellar FASI. The change in FASI size over time was not associated with any clinical symptoms or signs.

## Abbreviations

ADC: Apparent diffusion coefficients
FASI: Focal abnormal signal intensities
FLAIR: Fluid-attenuated inversion recovery images
FSPGR: Fast spoiled gradient echo images
NF1: Neurofibromatosis type 1
UBO: Unidentified bright objects
